# Ten-year follow-up of infliximab treatment for uveitis in Behçet disease patients: A multicenter retrospective study

**DOI:** 10.3389/fmed.2023.1095423

**Published:** 2023-01-20

**Authors:** Masaru Takeuchi, Yoshihiko Usui, Kenichi Namba, Hiroshi Keino, Masaki Takeuchi, Hiroshi Takase, Koju Kamoi, Keitaro Hase, Takako Ito, Kei Nakai, Kazuichi Maruyama, Eri Kobayashi, Hisashi Mashimo, Tomohito Sato, Nobuyuki Ohguro, Junko Hori, Annabelle A. Okada, Koh-hei Sonoda, Nobuhisa Mizuki, Hiroshi Goto

**Affiliations:** ^1^Department of Ophthalmology, National Defense Medical College, Tokorozawa, Japan; ^2^Department of Ophthalmology, Tokyo Medical University, Tokyo, Japan; ^3^Department of Ophthalmology, Faculty of Medicine and Graduate School of Medicine, Hokkaido University, Sapporo, Japan; ^4^Department of Ophthalmology, Kyorin University School of Medicine, Mitaka, Japan; ^5^Department of Ophthalmology, Yokohama City University Graduate School of Medicine, Yokohama, Japan; ^6^Department of Ophthalmology and Visual Science, Tokyo Medical and Dental University, Tokyo, Japan; ^7^Department of Ophthalmology, Graduate School of Medical Science, Kyushu University, Fukuoka, Japan; ^8^Department of Ophthalmology, Yodogawa Christian Hospital, Osaka, Japan; ^9^Department of Vision Informatics, Graduate School of Medicine, Osaka University, Suita, Japan; ^10^Department of Ophthalmology, Nippon Medical School, Tokyo, Japan; ^11^Department of Ophthalmology, Japan Community Health Care Organization Hospital, Osaka, Japan

**Keywords:** Behçet disease, uveitis, infliximab, biologics, Behçet uveitis, endogenous uveitis, non-infectious uveitis

## Abstract

**Purpose:**

To evaluate 10-year outcome of infliximab (IFX) treatment for uveitis in Behçet disease (BD) patients using a standardized follow-up protocol.

**Design:**

Retrospective longitudinal cohort study.

**Participants:**

140 BD uveitis patients treated with IFX enrolled in our previous study.

**Methods:**

Medical records were reviewed for demographic information, duration of IFX treatment, number of ocular attacks before IFX initiation, best corrected visual acuity (VA) at baseline and 1, 2, 3, 4, 5, and 10 years after IFX initiation, uveitis recurrence after IFX initiation and main anatomical site, concomitant therapies, and adverse events (AEs).

**Main outcome measures:**

10-year IFX continuation rate and change in LogMAR VA.

**Results:**

Of 140 BD patients, 106 (75.7%) continued IFX treatment for 10 years. LogMAR VA improved gradually after initiation of IFX, and the improvement reached statistical significance from 2 years of treatment. Thereafter, significant improvement compared with baseline was maintained until 10 years, despite a slight deterioration of logMAR VA from 5 years. However, eyes with worse baseline decimal VA < 0.1 showed no significant improvement from baseline to 10 years. Uveitis recurred after IFX initiation in 50 patients (recurrence group) and did not recur in 56 (non-recurrence group). Ocular attacks/year before IFX initiation was significantly higher in the recurrence group (2.82 ± 3.81) than in the non-recurrence group (1.84 ± 1.78). In the recurrence group, uveitis recurred within 1 year in 58% and within 2 years in 74%. Seventeen patients (34%) had recurrent anterior uveitis, 17 (34%) had posterior uveitis, and 16 (32%) had panuveitis, with no significant difference in VA outcome. In addition, logMAR VA at 10 years did not differ between the recurrence and non-recurrence groups. AEs occurred among 43 patients (30.7%), and 24 (17.1%) resulted in IFX discontinuation before 10 years.

**Conclusions:**

Among BD patients with uveitis who initiated IFX, approximately 75% continued treatment for 10 years, and their VA improved significantly and was maintained for 10 years. Uveitis recurred in one-half of the patients, but visual acuity did not differ significantly from the patients without recurrence.

## Introduction

Behçet’s disease (BD) is a chronic multisystem disease with primary manifestations of recurrent oral and genital aphthous ulcers, skin lesions, and sight-threatening uveitis (1, 2). Ocular involvement presents predominantly as bilateral panuveitis, occurring in 60–80% of BD patients (3). The clinical course of uveitis in BD patients is characterized by an acute, recurrent, non-granulomatous panuveitis associated with occlusive necrotizing retinal vasculitis compromising both arteries and veins (3). Repeated ocular attacks and underlying retinal vasculitis cause irreversible ocular tissue damage, resulting in extensive retinal atrophy, macular degeneration, and optic nerve atrophy.

BD-associated uveitis is still a leading cause of blindness worldwide (4–7), although the treatment has undergone a paradigm shift with the introduction of tumor necrosis factor (TNF)-α inhibitors. According to the European League Against Rheumatism (EULAR) guidelines, TNF-α inhibitors are recommended for BD patients with uveitis resistant to colchicine, corticosteroids, and various immunomodulators (8). As a TNF inhibitor, Infliximab (IFX) was first used for the treatment of refractory uveitis in BD over 20 years ago (9), and is currently the most frequently administered agent for BD-associated uveitis in Japan. Accumulating evidence has demonstrated remarkable beneficial effects of IFX in the treatment of refractory BD-associated uveitis (9–20). However, including our previous study (21), only a limited number of multicenter clinical trials or studies on the use of IFX in a large number of BD-associated uveitis patients have been published (13, 15, 22, 23). Although a few recent studies showed the long-term visual outcome of BD patients with uveitis treated with IFX for 10 years (19, 24–26), they were monocentric studies and/or the numbers of patients were small.

We conducted a retrospective multicenter long-term observational study on a cohort of BD-associated uveitis patients who had been treated with IFX for more than 10 years, aiming to investigate the visual outcome, uveitis recurrence after initiation of IFX treatment, relationship between visual acuity and uveitis recurrence, and adverse events during a 10-year follow-up period.

## Patients and methods

### Study design and participants

This is a retrospective follow-up study of our previous report (21) in which a total of 164 BD uveitis patients treated with IFX were investigated at 11 tertiary uveitis facilities. The study was conducted according to the tenets of the Declaration of Helsinki and was approved (institutional review board: 4047) by the ethics committees of the National Defense Medical College Hospital and institutional review board of each of the other facilities. The study protocol was described to all human subjects by opt-out method, and written informed consent was waived by the ethics committees due to the retrospective nature of the study. BD was diagnosed according to the criteria established by the Behçet’s Disease Research Committee of Japan (27). The standard regimen of IFX treatment was intravenous infusion at a dose of 5 mg/kg body weight on weeks 0, 2, and 6, and then every 8 weeks.

### Data collection

Medical records were reviewed. Patient demographic information, duration of IFX treatment, numbers of ocular attacks before the initiation of IFX treatment, best corrected visual acuity (VA) at baseline and at 1, 2, 3, 4, 5 and 10 years after initiation of IFX treatment, recurrence of uveitis after IFX initiation and the main anatomical site of recurrence, concomitant agents, and adverse events (AEs) were collected. Since this is a retrospective study, “Ocular attacks” before IFX treatment and “Recurrence of uveitis” during IFX treatment had been determined by each clinician in routine clinical setting at multiple participating institutions without the established definition. The main anatomical site of uveitis recurrence was based on the most severe ocular inflammation observed in each patient. For instance, if anterior uveitis and panuveitis were observed at different inflammatory attacks, the case was classified as panuveitis. A questionnaire was filled out by the ophthalmologists who treated the patients investigated in each participating institution, and the data were collected and analyzed at National Defense Medical College. The data of some patients investigated in the present study have been reported previously by individual institutions.

### Outcomes

The primary endpoint was 10-year IFX continuation rate and changes of VA between baseline and different time point observations during follow-up. The secondary endpoints are as follows: (1) the impact of concomitant therapies; (2) recurrence rate of uveitis after IFX initiation; (3) period to recurrence of uveitis after IFX initiation; (4) comparison between the recurrence and non-recurrence groups; (5) comparison between the main anatomical types of recurrent uveitis; and (6) AEs. LogMAR VA were analyzed comparing baseline with 1, 2, 3, 4, 5, and 10 years after IFX treatment. Adverse events were monitored throughout the study and reported up to 10 years after initiation of IFX.

### Statistical analysis

Statistical analyses were performed using JMP Pro version 15 (Business Unit of SAS, Cary, NC). The normality of data was analyzed by Shapiro–Wilk test. Continuation rate of IFX treatment was performed using the Kaplan–Meier method and log-rank test. Changes in logMAR VA from baseline to various time points after IFX initiation were analyzed by Steel’s multiple comparison test. Comparison of logMAR VA between independent groups was analyzed by Mann–Whitney *U* test (2 groups) or Steel-Dwass test (more than 3 groups), and by chi square test for categorical variables. A *P* value less than 0.05 was considered statistically significant.

## Results

### Continuation rate of IFX treatment and the association of concomitant agents

**FIGURE 1:**
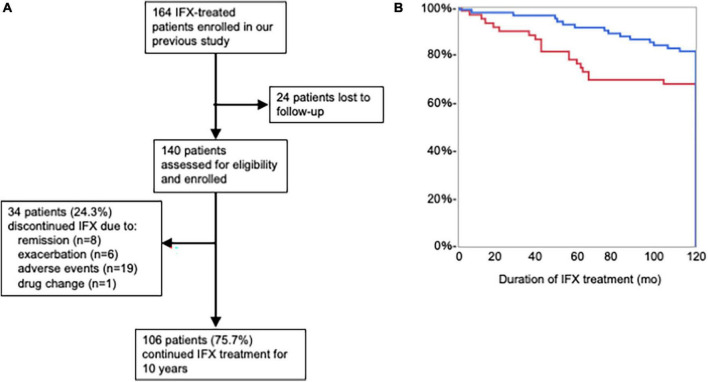
**(A)** In a total of 164 infliximab (IFX)-treated BD patients with uveitis, 24 patients were lost to follow-up after the initial study. Of the 140 BD patients included in the present analysis, IFX treatment was discontinued within 10 years in 34 patients (24.3%). One hundred six BD patients continued IFX treatment for more than 10 years, and 10-year IFX continuation rate was 75.7%. **(B)** The Kaplan–Meier curve of continuation rate of IFX treatment of BD uveitis patients with concomitant agents (red line) and those without concomitant agents (blue line). The IFX continuation rate more than 10 years was significantly higher in BD patients treated with IFX only (81.5%) than in those treated with IFX and concomitant agents (67.8%). log-rank test indicated *P* < 0.05.

Figure 1 shows disposition of BD uveitis patients with IFX treatment and the continuation rate of IFX treatment for 10 years with the association of concomitant agents. A total of 164 IFX-treated BD patients with uveitis were investigated in our previous study (21), but 24 patients were lost to follow-up after the initial study (Figure 1A). Of the 140 BD patients, 106 BD uveitis patients (75.7%) were continuing IFX treatment more than 10 years, but 34 patients (24.3%) discontinued the treatment within 10 years; due to remission of uveitis in 8 patients, relapse of uveitis in 6 patients, adverse events in 19 patients, and drug change in 1 patients.

The association of concomitant agents with IFX continuation rate is shown in Table 1 and Figure 1B. Concomitant agents were used in 40 of 106 BD patients (37.7%) who continued IFX treatment more 10 years, and in 19 of 34 BD patients (55.9%) who discontinued IFX treatment within 10 years. The IFX continuation rate more than 10 years was significantly higher in BD patients treated with IFX only (81.5%) than in those treated with IFX and concomitant agents (67.8%). Table 2 shows background of overall BD patients with uveitis and them classified into the continuous group who continued IFX treatment for more than 10 years and the discontinuous group who discontinued within 10 years. Mean age at initiation of IFX treatment was 47.3 ± 12.8 in overall, 46.3 ± 11.5 years in the continuous group, and 50.4 ± 16.2 years in the discontinuous group. Male to female ratio was 79: 21 in overall, 77: 23 in the continuous group, and 82: 18 in the discontinuous group. There were no age and sex differences between the continuous and discontinuous groups. Duration of IFX treatment was almost 3 times more in the continuous group (152.5 ± 13.9) than in the discontinuous group (52.6 ± 31.7). In concomitant agents, cyclosporin was more used in the discontinuation group (32.3%) than in the continuation group (13.2).

**TABLE 1:** Concomitant agents and 10-year infliximab continuation rate.

	IFX treatment; n (%)		IFX continuation rate (%)*	*P* value
	**Continuation**	**Discontinuation**		
IFX only	66 (62.3)	15 (44.1)	81.5	0.0414^†^
IFX + concomitant agents	40 (37.7)	19 (55.9)	67.8	
Total	106 (100)	34 (100)	75.7	

n, number of patients.

*10-year IFX continuation rate.

^†^Comparison of IFX continuation rate between IFX only and IFX + concomitant agents by log-rank test.

**TABLE 2:** Background of Behçet disease patients with uveitis who continued infliximab treatment for more than 10 years or discontinued within 10 years.

	Total (*n* = 140)	IFX treatment <10 yrs (*n* = 34)	IFX treatment ≧10 yrs (*n* = 106)	*P* value*
Age^†^, yrs Mean (SD)	47.3 (12.8)	50.4 (16.2)	46.3 (11.5)	0.1093
**Sex composition, no. (%)**				
Male	110 (78.6)	28 (82.4)	82 (77.4)	0.5369
Feale	30 (21.4)	6 (17.6)	24 (22.6)	
**Duration of IFX treatment, mo**				
Mean (SD)	128.2 (47.3)	52.6 (31.7)	152.5 (13.9)	<0.0001
Concomitant agents^‡^, *n.* (%)				0.0454
None	81 (57.9)	15 (44.1)	66 (62.3)	
Colchicine	33 (23.6)	7 (20.6)	26 (24.5)	
Corticosteroids	15 (10.7)	2 (5.9)	13 (12.2)	
Cyclosporine	25 (17.9)	11 (32.3)	14 (13.2)	
Methotrexate	2 (1.4)	0 (0)	2 (2.1)	
Azathioprine	2 (1.4)	0 (0)	2 (2.1)	

*Non-recurrence versus recurrence by Mann–Whitney U test for continuous variables and by chi square test for categorical variables.

^†^Age at the date of data collection.

^‡^Concomitant agents used with IFX, including overlap.

### Changes of LogMAR VA in BD uveitis patients treated with IFX for 10 years

**FIGURE 2:**
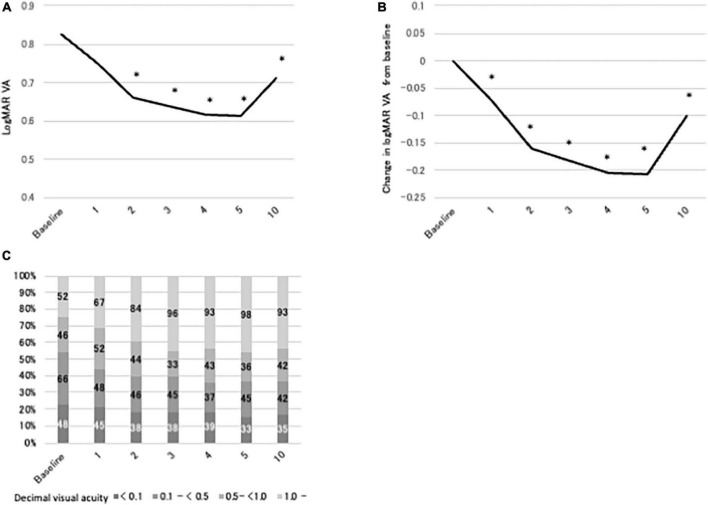
**(A)** Mean logMAR VA at baseline and at 1, 2, 3, 4, 5, and 10 years after initiation of infliximab treatment in all BD patients (*n* = 94). *P < 0.05 versus baseline by Steel’s multiple comparison test. **(B)** Mean change in logMAR VA from baseline at 1, 2, 3, 4, 5, and 10 years. *P < 0.05 versus baseline by Steel’s multiple comparison test. **(C)** Bar graph of proportions of eyes with different categories of VA at baseline and at 1, 2, 3, 4, 5, and 10 years. Chi square test indicated *P* = 0.0007.

**FIGURE 3:**
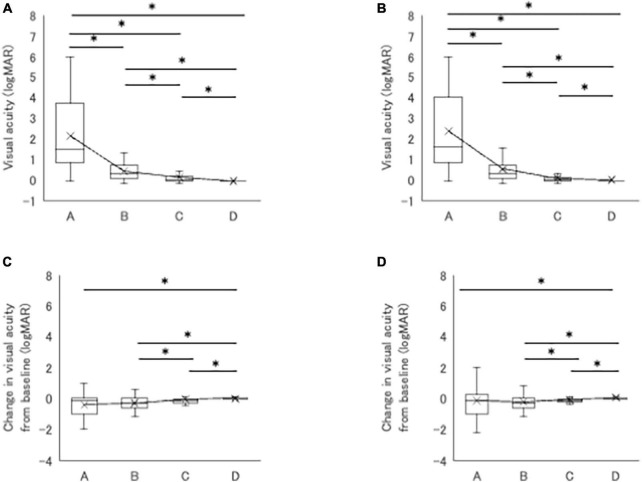
**(A,B)** Comparison of logMAR VA between four groups classified by VA at 5 **(A)** and 10 years **(B)**. Significant differences are observed between groups A, B, C, and D. **(C,D)** Comparison of changes in logMAR VA from baseline between four groups at 5 **(C)** and 10 years **(D)**. Significant differences are observed between group D and group A, B or C. Groups classified by VA: group A (<0.1, *n* = 42), group B (0.1 to <0.5, *n* = 57), group C (0.5 to <1.0, *n* = 40), and group D (≥1.0, *n* = 49). *P < 0.05 by Steel-Dwass test.

**FIGURE 4:**
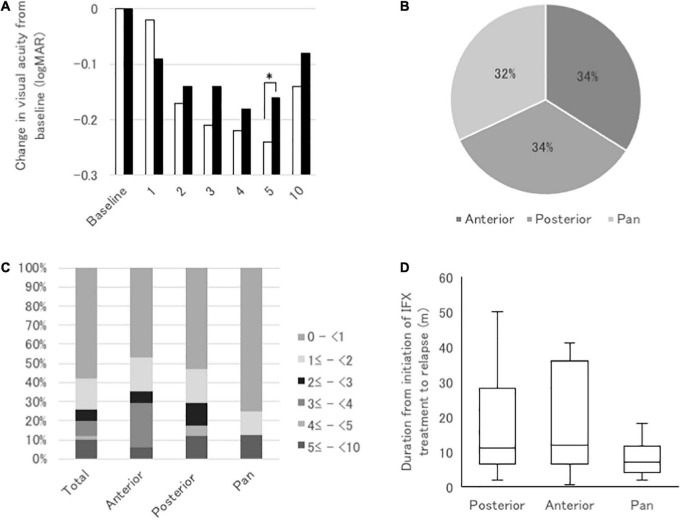
**(A)** Comparisons of mean changes in logMAR VA from baseline at 1, 2, 3, 4, 5, and 10 years after initiation of infliximab (IFX) between BD patients with uveitis recurrence after IFX initiation (closed bars) and those without recurrence (open bars). *P < 0.05 by Mann–Whitney *U* test. **(B)** Pie graph of proportions of recurrent uveitis classified by the main anatomical site of ocular inflammation; anterior uveitis, posterior uveitis, and panuveitis. **(C)** Bar graph of proportions of eyes with different durations from IFX initiation to uveitis relapse in three types of recurrent uveitis. **(D)** Box plot of duration from IFX initiation to uveitis relapse in three types of recurrent uveitis. *P < 0.05 by Steel-Dwass test.

Figure 2 shows logMAR VA, changes in logMAR VA from baseline, and distribution of VA at baseline and at 1, 2, 3, 4, 5, and 10 years after IFX initiation in the BD uveitis patients treated with IFX more than 10 years. LogMAR VA improved gradually after initiation of IFX, and the improvement reached statistical significance from 2 years of treatment (Figure 2A). Thereafter, significant improvement compared with baseline was maintained until 10 years, despite a slight deterioration of logMAR VA from 5 years. The same trend was observed in the plot of change in logMAR VA from baseline, except that significant improvement was observed earlier, from 1 year of treatment, and improved vision was maintained through 10 years. The distribution of decimal VA also changed significantly. The proportion of eyes with VA < 0.1 decreased from 23% at baseline to 17% at 10 years of treatment, and VA ≥ 1.0 increased from 25% at baseline to 44% at 10 years (Figure 2C). We next analyzed logMAR VA and changes in logMAR VA from baseline in 4 groups classified by decimal VA at baseline; group A (<0.1; *n* = 48), group B (0.1 to <0.5; *n* = 66), group C (0.5 to <1.0; *n* = 46), and group D (≥1.0; *n* = 52). The results are shown in Table 3. In group A, logMAR VA did not differ significantly between baseline and 1, 2, 3, 4, 5 or 10 years, although change in logMAR VA improved significantly from 2 through 10 years of IFX treatment compared with baseline. On the other hand, in group B, significant improvement in logMAR VA and in change in logMAR VA were observed from 1 through 10 years compared with baseline. The results in group C were similar to group B, with significant improvement in logMAR VA and in change in logMAR VA from 2 through 10 years compared with baseline. In group D, there were no significant differences in either logMAR VA or change in logMAR VA throughout 10 years of IFX treatment compared with baseline. Figure 3 compares logMAR VA and changes in logMAR VA from baseline between groups A, B, C and D at 5 and 10 years. Significant differences in logMAR VA between groups A, B, C and D remained at both 5 and 10 years (Figures 4A, B), although change in logMAR VA was the greatest in groups A followed by group B, group C, and group D at both 5 and 10 years (Figures 4C, D).

**TABLE 3:** LogMAR VA and changes in logMAR VA from baseline during 10 years after initiation of IFX treatment in eyes classified by decimal VA at baseline.

		Mean (SD)	*P* value*	Mean (SD)	*P* value*
A (*n* = 48)	Baseline	2.524		0	
		1.813		0	
	1	2.367	0.8206	−0.157	0.1030
		2.030		1.092	
	2	2.220	0.5030	−0.304	0.0012
		1.958		0.744	
	3	2.228	0.4955	−0.296	0.0036
		1.944		0.905	
	4	2.101	0..2568	−0.423	0.0019
		1.927		0.893	
	5	2.115	0.2212	−0.409	0.0036
		1.970		0.893	
	10	2.365	0.4321	−0.159	0.0160
		2.083		1.226	
		**Mean (SD)**	***P*** **value**	**Mean (SD)**	***P*** **value**
B (*n* = 66)	Baseline	0.729		0	
		0.216		0	
	1	0.585	0.0014	−0.144	<0.0001
		0.585		0.587	
	2	0.452	<0.0001	−0.277	<0.0001
		0.408		0.428	
	3	0.427	<0.0001	−0.302	<0.0001
		0.430		0.430	
	4	0.456	<0.0001	−0.273	<0.0001
		0.602		0.590	
	5	0.421	<0.0001	−0.308	<0.0001
		0.589		0.585	
	10	0.510	<0.0001	−0.219	<0.0001
		0.912		0.919	
C (*n* = 46)	Baseline	0.171		0	
		0.100		0	
	1	0.255	0.7254	0.084	0.1772
		0.459		0.455	
	2	0.152	0.0036	−0.019	<0.0001
		0.388		0.400	
	3	0.100	0.0008	−0.071	<0.0001
		0.274		0.282	
	4	0.083	<0.0001	−0.088	<0.0001
		0.319		0.326	
	5	0.098	<0.0001	−0.074	<0.0001
		0.314		0.324	
	10	0.076	<0.0001	−0.095	<0.0001
		0.292		0.294	
D (*n* = 52)	Baseline	−0.114		0	
		0.07		0	
	1	−0.080	0.4656	0.034	0.1340
		0.110		0.103	
	2	−0.110	1.000	0.004	0.9912
		0.06		0.072	
	3	−0.129	0.7644	−0.015	0.2381
		0.073		0.078	
	4	−0.124	0.8186	−0.010	0.8556
		0.071		0.008	
	5	−0.117	0.9963	−0.003	0.8556
		0.065		0.071	
	10	−0.059	0.9040	0.056	0.5581
		0.1202		0.204	

Groups classified by decimal VA at baseline; A, <0.1; B, 0.1 to <0.5; C, 0.5 to <1.0; and D, ≥1.0.

*Versus baseline by Steel’s multiple comparison test.

### Comparison of BD uveitis patients with or without recurrence of uveitis after infliximab treatment initiation

Of 106 patients, uveitis recurred after initiation of IFX treatment in 50 patients (recurrence group) and did not recur in 56 patients (non-recurrence group). Characteristics of BD uveitis patients of recurrence and non-recurrence group are show in Table 4. Mean age at initiation of IFX treatment was 47.0 ± 12.3 years (range 24–78 years) in the non-recurrence group and 45.7 ± 10.7 years (range 22–72 years) in the recurrence group. Male to female ratio was 45: 11 in the non-recurrence group and 37: 13 in the recurrence group. There were no age and sex differences between the recurrence and non-recurrence groups. In 212 eyes of 106 patients, the number of ocular attacks per year before IFX treatment was significantly higher in the recurrence group (2.82 ± 3.81) than in the non-recurrence group (1.84 ± 1.78). Mean logMAR VA at baseline (before IFX treatment) was 0.90 ± 1.40 and mean duration of IFX treatment was 150.6 ± 14.5 months in the non-recurrence group, while the corresponding values were 0.70 ± 1.20 and 154.6 ± 13.0 months in the recurrence group, with no significant differences between two groups. Regarding concomitant agents used with IFX (including overlap), colchicine was apparently more frequently used and corticosteroids and cyclosporine less frequently used in the non-recurrence group than in the recurrence group, but there were no significant differences.

**TABLE 4:** Characteristics of Behçet disease uveitis patients with or without recurrence of uveitis after infliximab treatment initiation.

	Non-recurrence (*n* = 56)	Recurrence (*n* = 50)	*P* value*
**Age^†^, yrs**			
Mean (SD)	47.0 (12.3)	45.7 (10.7)	0.5629
Median (range)	47 (24 – 78)	47 (22 – 72)	
**Sex composition, no. (%)**			
Male	45 (80.4)	37 (74.0)	0.4350
Female	11 (19.6)	13 (26.0)	
**Ocular attacks per year before IFX initiation^†^**			
Mean (SD)	1.84 (1.78)	2.82 (3.81)	0.0210
**BCVA at baseline of IFX treatment, logMAR**			
Mean (SD)	0.902 (1.404)	0.700 (1.197)	0.1052
**Duration of IFX treatment, months**			
Mean (SD)	150.6 (14.5)	154.6 (13.0)	0.2181
**Concomitant agents^‡^, no. (%)**			
None	36 (64.3)	30 (60.0)	0.2405
Colchicine	17 (30.4)	9 (18.0)	
Corticosteroids	4 (8.7)	9 (18.0)	
Cyclosporine	4 (8.7)	11 (22.0)	
Methotrexate	1 (2.2)	0	
Azathioprine	1 (2.2)	1 (2.0)	

*Non-recurrence versus recurrence by Mann–Whitney U test for continuous variables and by chi square test for categorical variables.

^†^Age at the date of data collection.

^‡^Concomitant agents used with IFX, including overlap.

Table 5 shows logMAR VA and changes in logMAR VA from baseline in the non-recurrence group and the recurrence group. Significant improvement in logMAR VA compared with baseline (0.90 ± 1.40) was observed from 2 (0.73 ± 1.48) through 10 years of IFX treatment (0.77 ± 1.60) in the non-recurrence group, but no significant change compared with baseline was found in the recurrence group throughout 10 years of IFX treatment. On the other hand, change in logMAR VA from baseline improved significantly from 1 through 10 years in the non-recurrence group and from 2 through 10 years in the recurrence group. Since logMAR VA at baseline was greater in the non-recurrence group (0.90 ± 1.40) than in the recurrence group (0.70 ± 1.20), we compared the changes in logMAR VA from baseline between the non-recurrence and the recurrence groups (Figure 4A). Significant greater improvement was observed in the non-recurrence group compared with the recurrence group at 5 years, but there was no significant difference between the two groups at 10 years.

**TABLE 5:** LogMAR VA and changes in logMAR VA from baseline during 10 years after initiation of IFX treatment in recurrence and non-recurrence groups.

	Duration of IFX treatment	LogMAR VA		Change in logMAR VA from baseline	
		**Mean SD**	***P*** value*	**Mean SD**	***P*** value*
Non-recurrence	Baseline	0.902		0	
		1.404		0	
	1	0.884	0.5480	−0.018	0.0017
		1.565		0.751	
	2	0.732	0.0287	−0.170	<0.0001
		1.476		0.508	
	3	0.688	0.0114	−0.214	<0.0001
		1.431		0.537	
	4	0.680	0.0067	−0.222	<0.0001
		1.429		0.606	
	5	0.658	0.0016	−0.244	<0.0001
		1.446		0.619	
	10	0.769	0.0060	−0.140	<0.0001
		1.604		0.828	
Recurrence	Baseline	0.700		0	
		1.197		0	
	1	0.608	0.9528	−0.092	0.1743
		1.121		0.529	
	2	0.556	0.6476	−0.144	0.0014
		1.086		0.460	
	3	0.559	0.3312	−0.141	<0.0001
		1.161		0.509	
	4	0.521	0.1936	−0.179	<0.0001
		1.119		0.522	
	5	0.540	0.2985	−0.160	0.001
		1.135		0.526	
	10	0.623	0.4940	−0.077	0.0017
		1.604		0.828	

*Versus baseline by Steel’s multiple comparison test.

**FIGURE 5:**
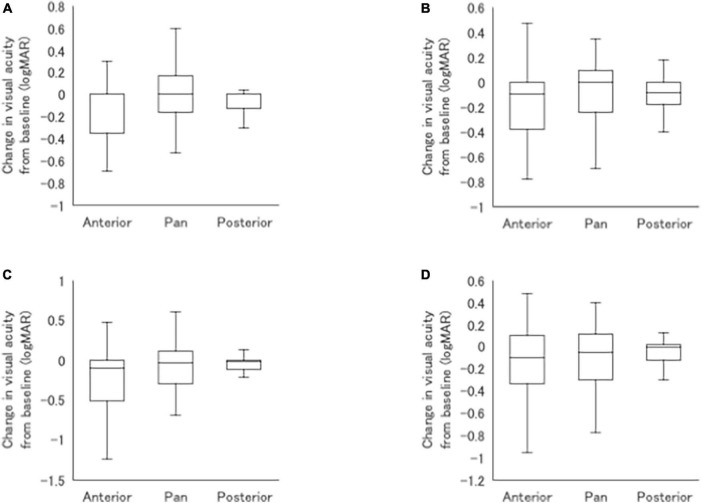
Comparisons of mean changes in logMAR VA from baseline between patients with recurrent anterior uveitis, posterior uveitis, and panuveitis at 1 **(A)**, 3 **(B)**, 5 **(C)**, and 10 **(D)** years after initiation of infliximab.

In the recurrence group, recurrent uveitis classified by the main anatomical inflammation site was anterior uveitis in 17 cases (34%), posterior uveitis in 17 cases (34%), and panuveitis in 16 cases (32%), with similar frequencies (Figure 4B). Uveitis recurred within 1 year of treatment in 58% of cases, and within 2 years in 74%. Posterior uveitis apparently recurred earlier than anterior uveitis, and panuveitis recurred earlier than posterior uveitis (Figure 4C), although there were no significant differences (Figure 4D). Figure 5 presents the changes in logMAR VA from baseline in eyes with recurrent anterior uveitis, posterior uveitis, and panuveitis at 1, 3, 5 and 10 years. There were no significant differences between the three groups at all the time points.

Table 6 shows the AEs observed in 140 BD patients with uveitis treated with IFX. AEs occurred in 43 BD patients (30.7%), 24 (17.1%) of whom discontinued IFX treatment within 10 years and 19 (13.6%) continued the treatment for more than 10 years. In 24 patients who discontinued IFX treatment, AEs included 12 cases (50%) of infusion reaction, 2 cases (8.3%) of pneumonia, 1 case (4.2%) each of tuberculosis, psoriasis, gastroenteritis, and liver damage. In 19 patients who continued IFX treatment, AEs included 10 cases (52.6%) of infusion reaction, 2 cases (10.5%) of eruption, and 1 case (5.3%) each of upper respiratory tract infection and gastroenteritis.

**TABLE 6:** Adverse events after IFX treatment.

	N (%)
Adverse events	43 (30.7)
Discontinued IFX	24 (17.1)
Infusion reaction	12 (50.0*)
Pneumonia	2 (8.3)
Tuberculosis	1 (4.2)
Psoriasis	1 (4.2)
Gastroenteritis	1 (4.2)
Liver disorder	1 (4.2)
Others	6 (25.0)
Continued IFX	19 (13.6)
Infusion reaction	10 (52.6^†^)
Eruption	2 (10.5)
Upper respiratory tract infection	1 (5.3)
Gastroenteritis	1 (5.3)
Others	5 (26.3)

*Percentages among discontinued IFX.

^†^Percentages among continued IFX.

## Discussion

In BD uveitis patients, repeated ocular attacks and underlying retinal vasculitis cause irreversible visual acuity impairment. In earlier reports on visual outcome prior to the use of TNF-α inhibitors for the treatment of refractory BD uveitis, 20–74% of patients became legally blind with visual acuity of 20/200 or less within 10 years after the onset of uveitis (6, 7, 28–30). As indicated by our present study and other reports investigating the visual acuity of BD patients with uveitis treated with IFX for more than 10 years (24–26), IFX treatment has greatly improved the visual prognosis of BD-related uveitis. VA worsens gradually from the onset of uveitis, but continued IFX treatment reverses the trend of vision loss. The present multicenter study indicated that logMAR VA improved significantly compared with baseline by IFX treatment, and the improved visual acuity was maintained up to 10 years, despite a slight non-significant deterioration at 10 years. On the other hand, we newly indicated that in the subanalysis comparing the non-recurrence group and the recurrence group during IFX treatment for 10 years, significant improvement in logMAR VA change from baseline was observed in both groups, although ocular attacks per year before IFX treatment was significantly more frequent in the recurrence group than in the non-recurrence group. It is conceivable that IFX treatment suppressed recurrent uveitis to the extent that visual acuity is not affected even in the recurrence group. In this study, approximately 80% of recurrent uveitis occurred within 2 years after initiation of IFX treatment, which is consistent with previous reports (24, 31–33). Regarding the duration from IFX initiation to uveitis recurrence, panuveitis was the shortest followed by posterior uveitis and anterior uveitis. However, the changes in logMAR VA from baseline at 1, 3, 5 and 10 years were not significantly different between eyes with recurrent anterior, posterior, and pan-uveitis, suggesting that the 3 types of recurrent uveitis probably do not differ in severity with respect to visual outcome.

The 10-year IFX continuation rates in BD patients with uveitis were 47.1% (25) and 70.3% (24) in two previous studies, and there were no differences between with and without concomitant agents in both reports. In the present study, although BD patients who discontinued IFX treatment in our previous study were excluded, the IFX continuation rate for 10 years was 75.7%, which was close to that in the latter report (24). However, the IFX continuation rate was significantly higher in patients not using concomitant agents (81.5%) than in those using concomitant agents (67.8%). The rate of concomitant agent use was significantly higher in IFX treatment discontinuation group (55.9%) than in the continuation group (37.7%), suggesting that BD patients discontinuing IFX treatment had a more severe course of uveitis requiring concomitant agents.

In BD patients with poor baseline visual acuity, IFX treatment does not achieve sufficient improvement in visual acuity (15, 16, 19, 21, 34). In the present study also, logMAR VA did not improve significantly after IFX treatment in BD patients with baseline VA < 0.1, although significant improvement in logMAR VA change was observed, indicating that even when IFX treatment improves visual acuity, the amount of improvement is limited. On the other hand, logMAR VA in BD patients with baseline VA ≥ 1.0 did not deteriorate after IFX treatment for 10 years, and mean logMAR VA was –0.06 ± 0.12. BD patients may benefit from IFX treatment on other ocular and extra-ocular manifestations, and in order to maintain good visual acuity for 10 years or longer, IFX treatment should be initiated before VA deteriorates to less than 1.0.

In our previous study also, significant improvement in logMAR VA compared to baseline was observed in BD patients with uveitis who received IFX treatment for 1 to 4 years, but not in the patients treated for more than 4 years in whom there was a longer period after the onset of BD uveitis before the introduction of IFX treatment (21). Keino et al. also reported that initiation of IFX therapy in BD patients within 18 months of uveoretinitis onset was more effective in maintaining good VA than after 18 months (26). It is possible that even when severe ocular inflammation is not clinically observed, subclinical retinal vasculitis may persist causing irreversible retinal tissue damage. In future study, we intend to evaluate the fluorescein fundus angiography findings of BD patients with uveitis before and after IFX treatment and the association with detailed optical coherence tomography findings and visual acuity.

AEs related to IFX treatment are expected to increase with continuous use of IFX and prolonged follow-up. A total of 43 AEs occurred among 140 patients (AE rate, 30.7%). When severe AE is defined as an event that leads to discontinuation of IFX treatment, 24 severe AEs were recorded (17.1%). The severe AE rate in this study is higher than the rates reported by earlier studies with shorter-term IFX treatment in BD patients with uveitis (12, 13, 15–18, 22, 23, 31). However, in the report by Horiguchi et al. who followed BD patients with uveitis treated with IFX for 10 years, the incidence of severe AEs responsible for discontinuation of IFX treatment was 22.2%, which is comparable to our report (24).

In Japan, the domestic diagnostic criteria for BD (27) but not the international diagnostic criteria (35) is still widely used in Japan. In both diagnostic criteria, oral ulcer, skin lesion, genital ulcer, and uveitis are 4 major symptoms, and the 3 major symptoms are required for diagnosis of BD. The difference is that the former must include oral ulcer among the 3 major symptoms for diagnosis of BD, and the pathergy test can be used as one of the major symptoms in the diagnosis. However, the clinical features of uveitis have not been defined by the either diagnostic criteria. The reason why the domestic diagnostic criteria are still widely used in Japan may be that there are not few patients with BD in Japan who have vascular lesions, intestinal lesions, arthritis, and/or central nervous system lesions, and these lesions are defined as minor symptoms in the Japanese diagnostic criteria but not in the international diagnostic criteria.

The limitations of the present study include the retrospective design and inclusion of only Japanese participants. Differences in institution, clinical practice, and clinician experience are potential causes of recording bias, selection bias, and treatment bias, as subjects were treated by different clinicians in routine clinical setting at multiple participating institutions. In addition, the questionnaire used to collect patient clinical data provided limited information, lacking systemic complications, ocular inflammation activity at baseline, ocular complications responsible for visual impairment, such as cataract, secondary glaucoma, cystoid macular edema, epiretinal membrane, macular degeneration, and optic nerve atrophy, detailed clinical course of IFX treatment, and AE management.

This study included BD patients who had already developed BD uveitis and had irreversible visual impairment before IFX treatment was approved in Japan. Currently, TNF inhibitors are being introduced before irreversible visual impairment occurs, and patients with primary or secondary failure of IFX are switched to adalimumab (ADA), and in the case of ADA, to IFX. In addition, since Golimumab which is one of other TNF inhibitors (36), and Anakinra and Canakinumab which are Interleukin (IL)-1 Inhibitors (37), are progressing to be used as new treatments for BD uveitis, it is expected further improving the visual prognosis of BD patients with uveitis.

In conclusion, approximately 75% of BD patients with uveitis continued IFX treatment for 10 years or longer, and the 10-year continuation rate was significantly higher in patients not treated with concomitant medications than in those treated with co-medications. Approximately 25% of the patients discontinued IFX treatment due to AEs, uveitis exacerbation, or uveitis quiescence. Visual acuity improved significantly compared with baseline by IFX treatment and improvement was maintained for 10 years, despite a slight non-significant deterioration after 5 years. Uveitis recurred after IFX treatment in approximately one-half of the patients. Although ocular attacks before IFX treatment were more frequent in the recurrence group than in the non-recurrence group, visual acuity improvement was not significantly different between the two groups at 10 years. The frequencies of recurrent anterior uveitis, posterior uveitis, and panuveitis were similar, and there was no significant difference in visual acuity outcome. However, eyes with worse baseline VA showed limited improvement in visual acuity, suggesting that IFX treatment should be initiated before VA deteriorates, in order to achieve and maintain good visual acuity for more than 10 years.

## Data availability statement

The raw data supporting the conclusions of this article will be made available by the authors, without undue reservation.

## Ethics statement

This study was conducted according to the tenets of the Declaration of Helsinki and was approved (Institutional Review Board: 4047) by the Ethics Committees of the National Defense Medical College Hospital and institutional review board of each of the other facilities. Written informed consent for participation was not required for this study in accordance with the national legislation and the institutional requirements.

## Author contributions

MRT: research design, manuscript preparation. YU, KNM, HK, MKT, HT, KK, KH, TI, KNK, KM, EK, HM, TS, NO, JH, AO, K-HS, NM, and HG: data acquisition and/or research execution. MRT and TS: data analysis and/or interpretation. All authors contributed to the article and approved the submitted version.
